# UMLF-COVID: an unsupervised meta-learning model specifically designed to identify X-ray images of COVID-19 patients

**DOI:** 10.1186/s12880-021-00704-2

**Published:** 2021-11-22

**Authors:** Rui Miao, Xin Dong, Sheng-Li Xie, Yong Liang, Sio-Long Lo

**Affiliations:** 1 Institute of Systems Engineering, Macau University of Science and Technology, Avenida Wai Long, Taipa, Macau China; 2 Guangdong-Hong Kong-Macao Joint Laboratory for Smart Discrete Manufacturing, Guangzhou, 510006 China; 3 Department of State Key Laboratory of Quality Research in Chinese Medicines, Macau University of Science and Technology, Avenida Wai Long, Taipa, Macau China; 4 Present Address: Faculty of Information Technology, Macau University of Science and Technology, Avenida Wai Long, Taipa, Macau China

**Keywords:** COVID-19, X-ray, CNN, UMLF-COVID

## Abstract

**Background:**

With the rapid spread of COVID-19 worldwide, quick screening for possible COVID-19 patients has become the focus of international researchers. Recently, many deep learning-based Computed Tomography (CT) image/X-ray image fast screening models for potential COVID-19 patients have been proposed. However, the existing models still have two main problems. First, most of the existing supervised models are based on pre-trained model parameters. The pre-training model needs to be constructed on a dataset with features similar to those in COVID-19 X-ray images, which limits the construction and use of the model. Second, the number of categories based on the X-ray dataset of COVID-19 and other pneumonia patients is usually imbalanced. In addition, the quality is difficult to distinguish, leading to non-ideal results with the existing model in the multi-class classification COVID-19 recognition task. Moreover, no researchers have proposed a COVID-19 X-ray image learning model based on unsupervised meta-learning.

**Methods:**

This paper first constructed an unsupervised meta-learning model for fast screening of COVID-19 patients (UMLF-COVID). This model does not require a pre-trained model, which solves the limitation problem of model construction, and the proposed unsupervised meta-learning framework solves the problem of sample imbalance and sample quality.

**Results:**

The UMLF-COVID model is tested on two real datasets, each of which builds a three-category and four-category model. And the experimental results show that the accuracy of the UMLF-COVID model is 3–10% higher than that of the existing models.

**Conclusion:**

In summary, we believe that the UMLF-COVID model is a good complement to COVID-19 X-ray fast screening models.

**Supplementary Information:**

The online version contains supplementary material available at 10.1186/s12880-021-00704-2.

## Background

Coronavirus disease 2019 (COVID-19) caused by SARS-CoV-2 has become one of the most serious epidemic diseases in the world since the twentieth century [1–3]. The main symptoms of COVID-19 include dry cough, sore throat, fever, organ failure, septic shock, severe pneumonia, acute respiratory distress syndrome (ARDS), etc. [3–5]. Due to the highly contagious nature of COVID-19, medical systems in many countries are on the verge of collapse [6]. To date, there remains no specific medicine for COVID-19. Therefore, patients can only clear the virus through their own immune systems [7], directly leading to the rapid increase in the death rate of COVID-19. Tens of thousands of have people died because of COVID-19 [2, 8]. In this situation, stopping the spread of the virus has become the focus of international researchers.

Researchers have proposed many methods to combat the COVID-19 pandemic [9–12]. However, previous studies have shown that the best way to stop the spread of COVID-19 is to screen people infected with COVID-19 as quickly as possible [13–15]. Currently, reverse transcription-polymerase chain reaction (RT–PCR) is the most commonly used diagnostic test for COVID-19 [16–18]. However, the sensitivity of the RT–PCR test is low, and the test time of RT–PCR is long in the early stage [19–22]. The cost of RT–PCR testing in hospitals is also high. Therefore, it is difficult for many countries to conduct large-scale nucleic acid testing. In this case, using CT images or X-ray images for rapid preliminary screening of subjects with potential pneumonia symptoms is a feasible solution to this problem [22, 23]. Many machine learning and deep learning methods have brought great help to the fight against COVID-19[24–27]. However, CT/X-ray images of COVID-19 are very similar to those of traditional pneumonia, which requires experienced experts to diagnose COVID-19 patients based on CT/X-ray images [28, 29]. Many regions cannot implement the program due to a lack of experts. Therefore, some COVID-19 potential patient detection models based on CT images have been proposed and have achieved good results [30–32]. Although CT images can provide better details than X-ray scans, CT scans cause more harm to the human body, and the cost of CT scanning is also high. Therefore, many researchers recommend using X-ray imaging instead of CT imaging for preliminary screening [33, 34]. X-ray imaging has the characteristics of fast speed, low cost and minor damage.

At present, researchers have established some X-ray image datasets of COVID-19 patients. For example, Mahmud et al. proposed CovXNet, which is a COVID-19 X-ray image detection model based on transfer learning [34]. Shorfuzzamana et al. proposed MetaCOVID, which is a supervised meta-learning model [35].

However, the existing models still have two main problems. First, most of the existing models are supervised. The initialization of these models is based on pre-training, and the dataset images used by the pre-trained model need to have features similar to those of COVID-19 X-ray images. These issues limit the construction of models. Second, the number of categories based on the X-ray dataset of COVID-19 and other pneumonia patients is usually imbalanced. The quality is also difficult to distinguish, making it difficult for the model to use the supervised meta-learning model directly for training. This issue also increases the difficulty of transfer learning and leads to non-ideal results with the model in the multi-class classification COVID-19 recognition task.

**Fig. 1:**
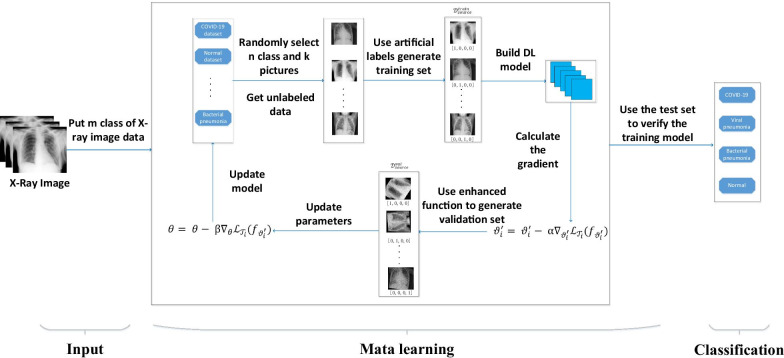
The flow chart of UMLF-COVID. The model requires multiple classes of images, which will randomly sample n-way k-shot images and attach artificial labels. The augmentation function is adopted to generate the validation dataset

This paper proposes an unsupervised meta-learning recognition model for COVID-19 X image detection (UMLF-COVID). The UMLF-COVID model does not require pre-trained model parameters, which solves the limitation problem of model construction. The proposed unsupervised meta-learning framework only needs to have few pneumonia pictures in each cycle, which solves the problem of sample imbalance and sample quality. An n-way k-shot training form and a gradient-based meta-learning optimization strategy are adopted in this paper. The UMLF-COVID model is unsupervised in the meta-learning step, which randomly samples K images for each N class and uses artificial labels to build a training set. This training set is related to the target but does not require proper category labels. Next, the model uses a validation dataset to update the gradient based on the deep learning model. Another feature of the model is that the validation set is created based on a training data sample using an enhancement function, which solves the limitation on the number of categories in the COVID-19 dataset (Fig. 1). The experiment uses two real datasets to test UMLF-COVID and constructs three-category and four-category models for each dataset individually. In addition, the model contains a 4-layers neural network. In the experimental results, the model can effectively identify COVID-19 patients and others. The UMLF-COVID model achieves a comprehensive recognition accuracy of 0.94 in the three-classification experiment (COVID-19, normal person, and other pneumonia). In the four-classification experiment (COVID-19, normal person, virus pneumonia, bacterial pneumonia), the model reached a recognition accuracy of 0.9.

The main contributions of this paper can be summarized as follows:

First, the UMLF-COVID model is the first approach based on unsupervised meta-learning to identify COVID-19 X-ray images.

Second, the UMLF-COVID model does not need pre-trained parameters, which solves the limitation problem of model construction.

Third, it does not have high requirements for the number of samples of each type of pneumonia, which solves the problem of small sample size and unbalanced COVID-19 X-ray data.

Fourth, according to the experimental results, the UMLF-COVID model is better than the existing supervised model. Because it can learn the experience of multiple pneumonia X-ray classification tasks during the training step, the UMLF-COVID model ultimately achieves better results on the fixed COVID-19 pneumonia classification task.

Fifth, two real datasets are set to verify the performance, and the experimental results show that the UMLF-COVID is better than existing models. At the same time, only a 4-layer neural network is needed to construct an outstanding prediction model.

## Methods

### Dataset

Because the number of patients diagnosed with COVID-19 is small. Therefore, only conducting an experiment on one data set is not enough to prove the performance of the model. In order to verify the performance and stability of the UMLF-COVID model. This paper obtained three available COVID-19 X-ray and other pneumonia datasets to construct two experimental datasets, and the description is as follows.

#### BIMCV-COVID19 + dataset

The BIMCV-COVID19 + dataset [36] is a large dataset that contains chest X-ray images (CXR) and Computed Tomography (CT) images of some COVID-19 patients. The dataset includes the demographic information of the patient and the label information of the image. The first version of this dataset includes 1,380 CX images, 885 DX images and 163 CT images.

#### Kaggle dataset

The fourth external validation was performed on an open public Kaggle-pneumonia dataset [37]. This dataset contains three folders (training, testing, validation), and each folder includes subfolders of the image category (viral pneumonia/bacterial pneumonia/normal). There were 5,663 chest X-ray images (front and back) collected from a retrospective study of paediatric patients aged 1–5 years in Guangzhou Maternal and Child Health Center. These images were first screened for quality control by removing low-quality or unreadable scans. Two professional physicians classified the diagnosis of the image and then cleared it to train the AI system. To resolve any grading errors, the dataset was also checked by another expert.

#### Chest XRay_AI dataset

De-identified and anonymized data were deposited into the China National Center for Bioinformation [38]. The chest X-ray image (CXR) dataset was constructed by the China Chest X-ray Image Investigation Association (CC-CXRI). This dataset can be used globally to help researchers study COVID-19. An AI model is first used to identify common chest diseases, including atelectasis, cardiac hypertrophy, consolidation, oedema, effusion, emphysema, fibrosis, hernia, infiltration, nodules, masses, pleural thickening, pneumonia and pneumothorax. ChestDX and ChestDx-PE are datasets of patients. The CC-CXRI-P dataset contains viral pneumonia (including COVID-19 pneumonia), other types of pneumonia and normal images.

**Fig. 2:**
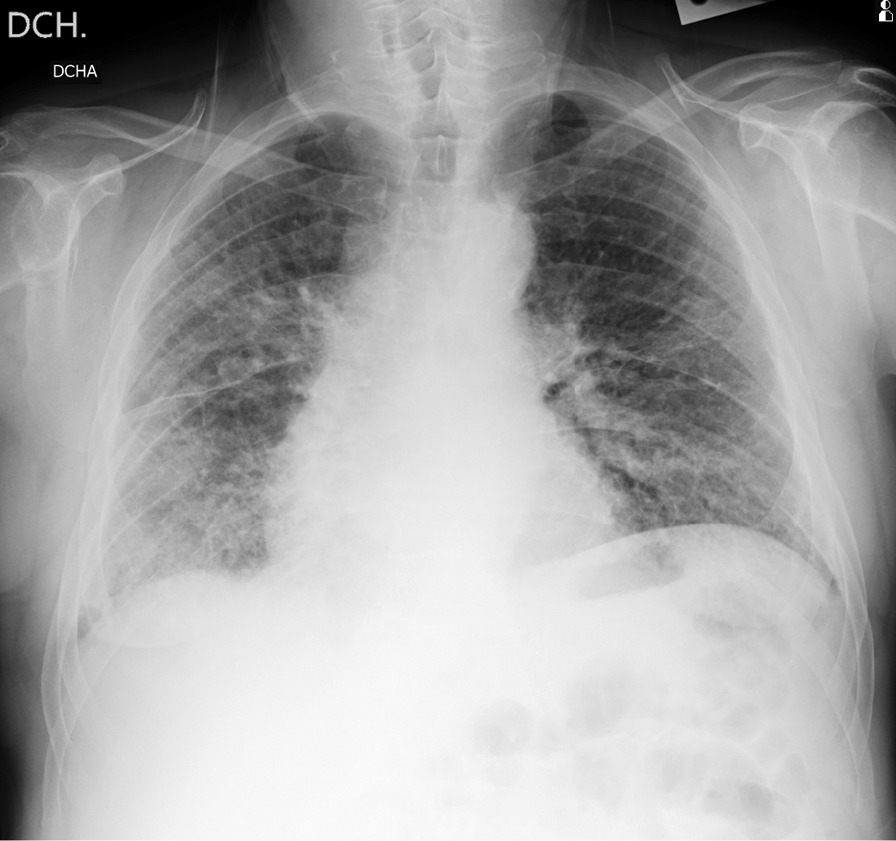
An example image of the BIMCV dataset identified as COVID-19 patients. The area of pneumonia infiltrated almost the entire right and left hemipleural cavities, mainly in the middle and basal areas, and no pleural effusion was seen. Assess possible COVID-19 patients in a clinical setting

These three public datasets are divided into two experimental datasets based on the random sampling method, namely, BIMCV and Xray_AI. The experimental dataset is shown in Table 1. The total number of X-ray images is 1,027, including 395 of COVID-19 patients and 632 of non-COVID-19 patients. Each experimental dataset contains five classification labels: COVID-19, normal person, bacterial pneumonia, viral pneumonia and other pneumonia. This paper only uses the frontal X-ray images of the chest to experiment (Fig. 2).

**Table 1:** The number of X-ray images in each experimental dataset

	COVID-19	Bacteria	Virus	Normal	Other pneumonia
BIMCV	395	1200	672	1104	630
XRay_AI	367	1269	600	1349	627

There are two datasets, BIMCV and XRay_AI

### Model

This paper resizing all the images in the dataset to the same size as the preliminary pre-processing step, making the subsequent processes faster and easier to fine-tune. The image size that this paper used is 500 × 500.

#### Meta-learning framework

The idea of meta-learning is to learn a general learning algorithm that can achieve good learning results in multiple tasks [39]. Ideally, the model can learn the common experience in different tasks, and in each new task, it can obtain a better result than the previous task. This is the reason why meta-learning is better than traditional single-task models, and the performance of $\omega$ can be evaluated on a task distribution $p\left( {\mathcal{T}} \right)$ for each task involving a dataset and a loss function $T = \left\{ {{\mathcal{D}},{\mathcal{L}}} \right\}$. Here, $\omega$ is a parameter indicating ‘how to study’, such as the optimizer that selects model parameter $\theta$. Therefore, the general model of meta-learning can be represented as Formula (1):

1 $$\mathop {\min }\limits_{\omega } \mathop {\mathbb{E}}\limits_{{{\mathcal{T}}\sim p\left( {\mathcal{T}} \right)}} {\mathcal{L}}\left( {{\mathcal{D}};\omega } \right)$$

where ${\mathcal{L}}$ is the loss of the task. While divide the training dataset into ${\mathcal{D}} = \left( {{\mathcal{D}}^{train} ,{\mathcal{D}}^{val} } \right)$. Then, the task-specific loss can be defined as Formula (2):

2 $${\mathcal{L}}\left( {{\mathcal{D}};{\upomega }} \right) = {\mathcal{L}}\left( {{\mathcal{D}}^{{{\text{val}}}} ;{\uptheta }^{*} \left( {{\mathcal{D}}^{{{\text{train}}}} ,{\upomega }} \right){\upomega }} \right)$$

where $\theta^{*}$ is the parameter trained in the $\omega$ model and ${\mathcal{D}}^{train}$.

Normally, assume a set of *M* source tasks are sampled from $p\left( {\mathcal{T}} \right)$. Denote the *M* source task set used in the meta-training stage as ${\mathcal{D}}_{source} = \left\{ {\left( {{\mathcal{D}}_{source}^{train} ,{\mathcal{D}}_{source}^{val} } \right)} \right\}_{1}^{M}$, where each task has training and verification data. The source training and validation datasets are called the supporting and query datasets, respectively. The meta-training step of $\omega$ can be written as Formula (3):

3 $$\omega^{*} = \mathop {\text{arg min}}\limits_{\omega } \mathop \sum \limits_{i = 1}^{M} {\mathcal{L}}\left( {{\mathcal{D}}_{source}^{i} ;\omega } \right)$$

Moreover, the target datasets used in the test step are denoted as ${\mathcal{D}}_{target} = \left\{ {\left( {{\mathcal{D}}_{target}^{train} ,{\mathcal{D}}_{target}^{test} } \right)} \right\}$, where the test dataset still contains training and test data. In the training step, the model uses the learned meta-knowledge $\omega^{*}$ to train and test the fixed task of the test set, and the optimization goal of the parameter $\theta$ can be written as Formula (4):

4 $$\theta^{*} = \mathop {\text{arg min}}\limits_{\theta } {\mathcal{L}}\left( {{\mathcal{D}}_{target}^{train} ;\theta ,\omega^{*} } \right)$$

Compared with the traditional supervised training model, the target task of the meta-learning method can benefit from meta-knowledge $\omega^{*}$, which can be the empirical information of multiple tasks. This makes it possible for the meta-learning method to achieve better training effects than traditional methods on the target task.

#### Construct training dataset

Assuming there is a dataset ${\mathcal{U}}$ that contains *N* classes and *M* samples, it can be denoted as Formula (5):

5 $${\mathcal{U}} = \left\{ {\left( {x_{1} ,1} \right),\left( {x_{2} ,1} \right), \ldots ,\left( {x_{M} ,N} \right)} \right\}$$

UMLF-COVID model performs meta-training with n-way k-shot classification. That is, the number of classifications of the classifier during training is n, and each category contains k images. To remove existing labels, the model randomly samples n*k images from the whole dataset and attaches artificial labels. However, it is important to keep the labels distinguished by classes in actual operation: if two images have the same labels, they should have the same artificial labels. Therefore, UMLF-COVID model adopt the following strategy to ensure label distinction.

First, the UMLF-COVID model randomly $n\left( {n \in N} \right)$ classes from dataset ${\mathcal{U}}$ to build a subset. Each class is regarded as a group and can be denoted by Formula (6):

6 $${\hat{\mathcal{U}}} = \left\{ {\left( {\left( {x_{1} ,1} \right) \ldots \left( {x_{100} ,1} \right)} \right), \ldots ,\left( {\left( {x_{h - 500} ,n} \right), \ldots ,\left( {x_{h} ,n} \right)} \right)} \right\}$$

where *h* is the number of images from selected classes. Then, the UMLF-COVID model shuffle the order of ${\hat{\mathcal{U}}}$ by group and remove labels to construct an unsupervised dataset, which can be denoted as Formula (7):

7 $${\hat{\mathcal{U}}} = \left\{ {\left( {\left( {x_{h} } \right), \ldots ,\left( {x_{h - 500} } \right)} \right), \ldots ,\left( {\left( {x_{100} } \right), \ldots ,\left( {x_{1} } \right)} \right)} \right\}$$

The UMLF-COVID model randomly select k images for each group and put them into the training set. The UMLF-COVID model can obtain an unlabelled metadata training set ${\mathcal{T}}$, which can be denoted as Formula (8):

8 $${\mathcal{T}} = \left\{ {x_{1} , \ldots x_{{n{*}k}} } \right\}$$

Attaching artificial labels to ${\mathcal{T}}$, the UMLF-COVID model obtain the training set ${\mathcal{D}}_{source}^{train}$ required by the model, which can be denoted as Formula (9):

9 $${\mathcal{D}}_{source}^{train} = \left\{ {\left( {x_{1} ,1} \right), \ldots ,(x_{{n{*}k}} ,n)} \right\}$$

Formula (7) ensures that the classes of the meta-training set extracted by the model will be disrupted every time, and artificial labels are given in order. Even if the model extrac n of the same classes, it may show different classification forms in the artificial labels. Formula (8) ensures the classification of artificial labels. After the above operations, the UMLF-COVID model can build an unsupervised training set of metadata for the model, given the classification information through artificial labels. The algorithm for constructing the training dataset is denoted as Algorithm 1.
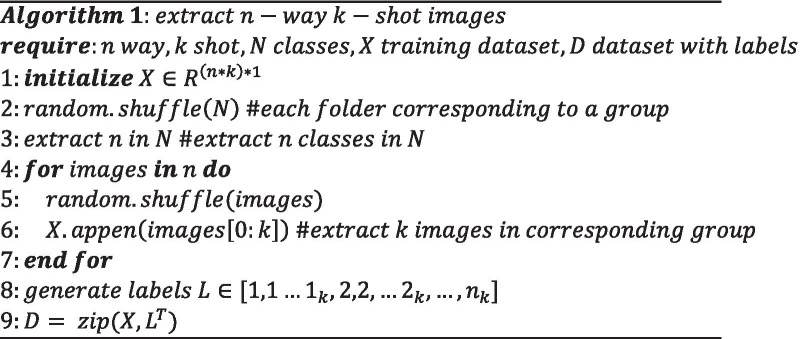


#### Construct validation datasets

Generally, the meta-learning model based on the gradient update principle requires the data distribution of the training dataset and validation datasets to be completely different, ensuring that the effect of gradient update is good enough. However, due to the limitation of the number of categories for the COVID-19 dataset, it is difficult to find a new class to build a validation dataset. The UMLF-COVID model adopt an enhancement function on the training set to construct a validation dataset to solve this problem. This paper believe that the distribution of the validation dataset generated by the enhancement function is different from that of the training dataset, which guarantees the effect of the gradient update. The final experimental results also verified this ideas.

The UMLF-COVID model use three kinds of enhancement functions: salt and pepper noise, Gaussian noise and random shift. One or more kinds of enhancement functions were used in the training set. The UMLF-COVID model can obtain a validation set ${\mathcal{D}}_{source}^{val}$, which can be denoted as Formula (10):

10 $${\mathcal{D}}_{source}^{val} = \{ \left( {{\mathbb{E}}(x_{1} } \right),1), \ldots ,({\mathbb{E}}(x_{{n{*}k}} ),n)\}$$

#### Update gradient

The goal of the UMLF-COVID model is to find a universal model in the multi-classification pneumonia dataset so that it can be quickly generalized to the three-class and four-class tasks of identifying COVID-19 X-ray images with only a small quantity of data. Therefore, the UMLF-COVID model also use the principle of gradient-based optimization to build a meta-learning model, and the loss function of the model is shown in Formula (11):

11 $${\mathcal{L}}\left( \emptyset \right) = \mathop \sum \limits_{n = 1}^{N} l^{n} \left( {\hat{\theta }^{n} } \right)$$

where $\emptyset$ denotes parameter of network. $\hat{\theta }^{n}$ is the parameter of the *n* sub-task learned based on $\emptyset$. $l^{n}$ is the loss function based on $\hat{\theta }^{n}$.

Because the task of the UMLF-COVID model is classification, the UMLF-COVID use cross-entropy as the loss function of the task, which can be denoted as Formula (12):

12 $${\mathcal{L}}_{{{\mathcal{T}}_{i} }} \left( {f_{\emptyset } } \right) = \mathop \sum \limits_{{x^{\left( j \right)} ,y^{\left( j \right)} \sim {\mathcal{T}}_{i} }} y^{\left( j \right)} logf_{\emptyset } \left( {x^{\left( j \right)} } \right) + \left( {1 - y^{\left( j \right)} } \right){\text{log}}\left( {1 - f_{\emptyset } \left( {x^{\left( j \right)} } \right)} \right)$$

where ${\mathcal{T}}_{i}$ is the i-th task and $x^{\left( j \right)} ,y^{\left( j \right)}$ are the input and output of ${\mathcal{T}}_{i}$. $f_{\emptyset }$ denotes the model function, which is determined by the parameter θ.

**Fig. 3:**
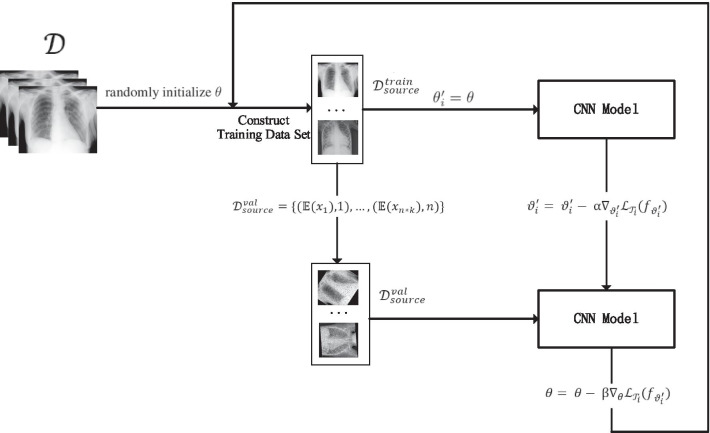
The details of the meta-learning step of the UMLF-COVID model

After establishing the loss function, the UMLF-COVID use the following method to update the gradient and set the initial parameters of the deep learning model in task ${\mathcal{T}}_{i}$ as $\theta$. The UMLF-COVID use Formula (13) to compute gradient (Fig. 3):

13 $$\vartheta_{i}^{\prime} = \vartheta_{i}^{\prime} - {{ \upalpha }}\nabla_{{\vartheta_{i}^{\prime} }} {\mathcal{L}}_{{{\mathcal{T}}_{i} }} \left( {f_{{\vartheta_{i}^{\prime} }} } \right)$$

where $\vartheta_{i}^{\prime} = \theta$. Then, the UMLF-COVID use a validation set ${\mathcal{D}^{\prime}}$ to update gradient $\theta$ as Formula (14):

14 $$\theta = \theta - {{ \upbeta }}\nabla_{\theta } {\mathcal{L}}_{{{\mathcal{T}}_{i} }} \left( {f_{{\vartheta_{i}^{\prime} }} } \right)$$

Finally, the UMLF-COVID algorithm can be denoted as Algorithm 2.
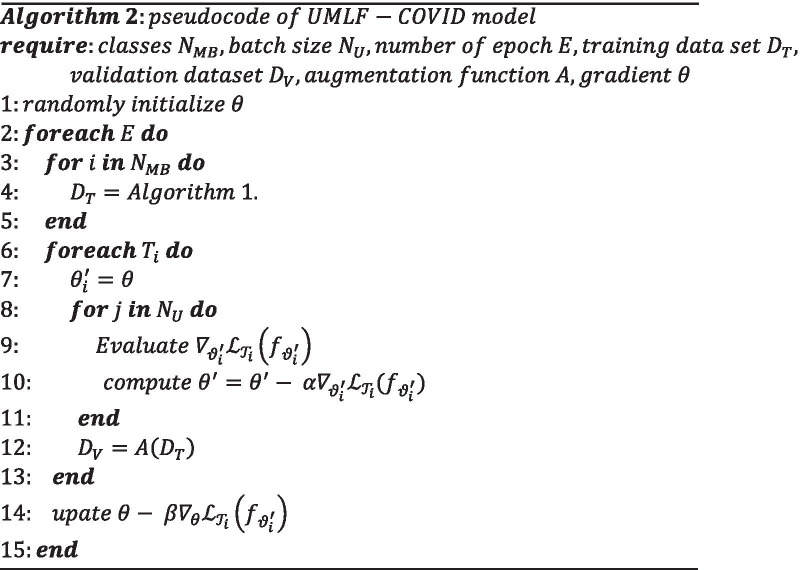


#### Parameters of deep learning

Based on the above-unsupervised learning framework, the UMLF-COVID construct a 4-layer neural network model containing 4-layer 2-dimensional convolution layers, and each convolution layer has max-pooling and a batch normalization layer.

**Fig. 4:**
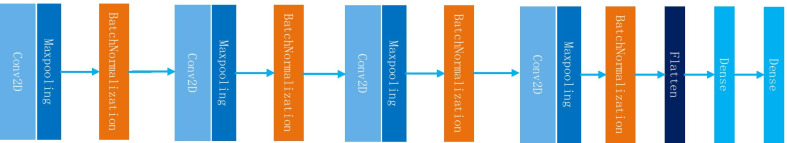
Model architecture. There are 4-layer 2-dimensional convolution layers, max-pooling layers and batch normalization layers

The LXCRM model uses a 3*3 convolution kernel. A small convolution kernel means fewer parameters and less time complexity. The first convolution layer uses 16 convolution kernels, the second uses 32 convolution layers, and the third uses 64 convolution layers (Fig. 4).

### Comparison models

#### LeNet5

LeNet-5 comes from the paper Gradient-Based Learning Applied to Document Recognition, which is a classic convolutional neural network [40] and is widely used in handwritten text recognition and other object classification applications. LeNet-5 is a simpler convolutional neural network. The main structure of LeNet-5 is as follows: the two-dimensional input image first passes through the convolutional layer twice to the pooling layer, then passes through the fully connected layer, and finally uses softmax classification as the output layer. The purpose of comparing the LeNet-5 model is to verify whether a simple deep learning model can effectively identify X-ray images of COVID-19 patients.

#### AlexNet

AlexNet was designed by Hinton and his student Alex Krizhevsky and won the 2012 ImageNet competition [41]. AlexNet is a classic deep learning architecture. The architecture contains 5 convolutional layers and three fully connected layers and uses the dropout layer. The original text uses 2 GPUs for training and limits the network size. Due to the improvement of hardware equipment, this paper only uses a GPU for computing.

#### VGG19

VGG was proposed by the Visual Geometry Group of Oxford. The network is related to the work on ILSVRC 2014 and shows that increasing the depth of the network can affect the performance to a certain extent. The improvement of VGG compared to AlexNet is using several consecutive 3 × 3 convolution kernels to replace the larger convolution kernel in AlexNet. A smaller convolution kernel reduces the number of model parameters and can better maintain the properties of the image. With the idea of the VGG model, the LXCRM model uses a 3 × 3 convolution kernel. VGG19 contains 19 hidden layers (16 convolutional layers and three fully connected layers). The purpose of comparing the VGG19 model is to verify whether the complex deep learning model can effectively identify X-ray images of COVID-19 patients.

#### CovXNet

CovXNet was proposed by Shorfuzzaman et al. and uses transfer learning and depthwise convolution with varying dilation rates to efficiently extract diversified features from chest X-rays [34]. In the training phase, the CovXNet model first uses a large database of normal people and non-COVID-19 patients for migration learning. Next, the trained model is migrated to a dataset containing COVID-19 patients to fine-tune the parameters. In the process of data reading, the CovXNet model uses convolution kernels of various sizes to integrate the local and global features of the image.

#### CNN-LSTM

In 2020, Islam et al. proposed a combination of a convolutional neural network (CNN) and long short-term memory (LSTM) with a deep learning model to automatically diagnose COVID-19 from X-ray images [42]. In this system, the CNN is used for deep feature extraction, and LSTM is used for detection using the extracted features.

#### CNN-RNN

The CNN-RNN model was used by RAKHAMI et al. in 2021 to quickly identify COVID-19 cases from chest X-rays. Similar to CNN-LSTM, the CNN is also used to extract features [43]. Then, the model uses the recurrent neural network (RNN) method to classify images.

#### EMCNet

In 2021, Saha et al. proposed the EMCNet model [44]. The model first uses the CNN to extract features from the chest X-ray image and then adds machine learning methods for image classification.

## Results

In this experiment, UMLF-COVID is constructed in JupyterLab with 32 GB RAM NVIDIA Tesla V100. The classification neural network is implemented by TensorFlow. The versions of Python and TensorFlow are 3.6.9 and 2.4.1, respectively. The experiment builds three-class and four-class classifiers for five models including comparison methods and adopts three strategies involving 1-shot, 5-shot, and 10-shot for each classifier in UMLF-COVID.

There are two datasets used in the experiment to test performance. This paper uses tenfold cross-validation for the experiment. For each model, the average results are reported in the following sections.

There are seven indicators used to evaluate the models: accuracy, precision, recall, F1-score, AUC using receiver operating characteristic (ROC) and precision-recall (PR). Accuracy is commonly used in deep learning, indicating the ratio of judgements for image classification. Due to the imbalance of the dataset, accuracy cannot express the performance of the model well. Therefore, this paper calculates the precision, recall, and F1-score indicators. Precision indicates the proportion of potential COVID-19 patients predicted to be COVID-19 potential patients. Recall is the proportion of potential COVID-19 patients who are correctly predicted. The PR curve is drawn based on the precision and recall values. The F1-score is a comprehensive manifestation of precision and recall indicators. Moreover, the ROC curve uses two parameters, the true positive rate and the false positive rate, to indicate the performance of classification tasks. The details of the comparison methods can be seen in the Additional file 2 and Additional file 1: Table S1.

### Results of BIMCV dataset

**Fig. 5:**
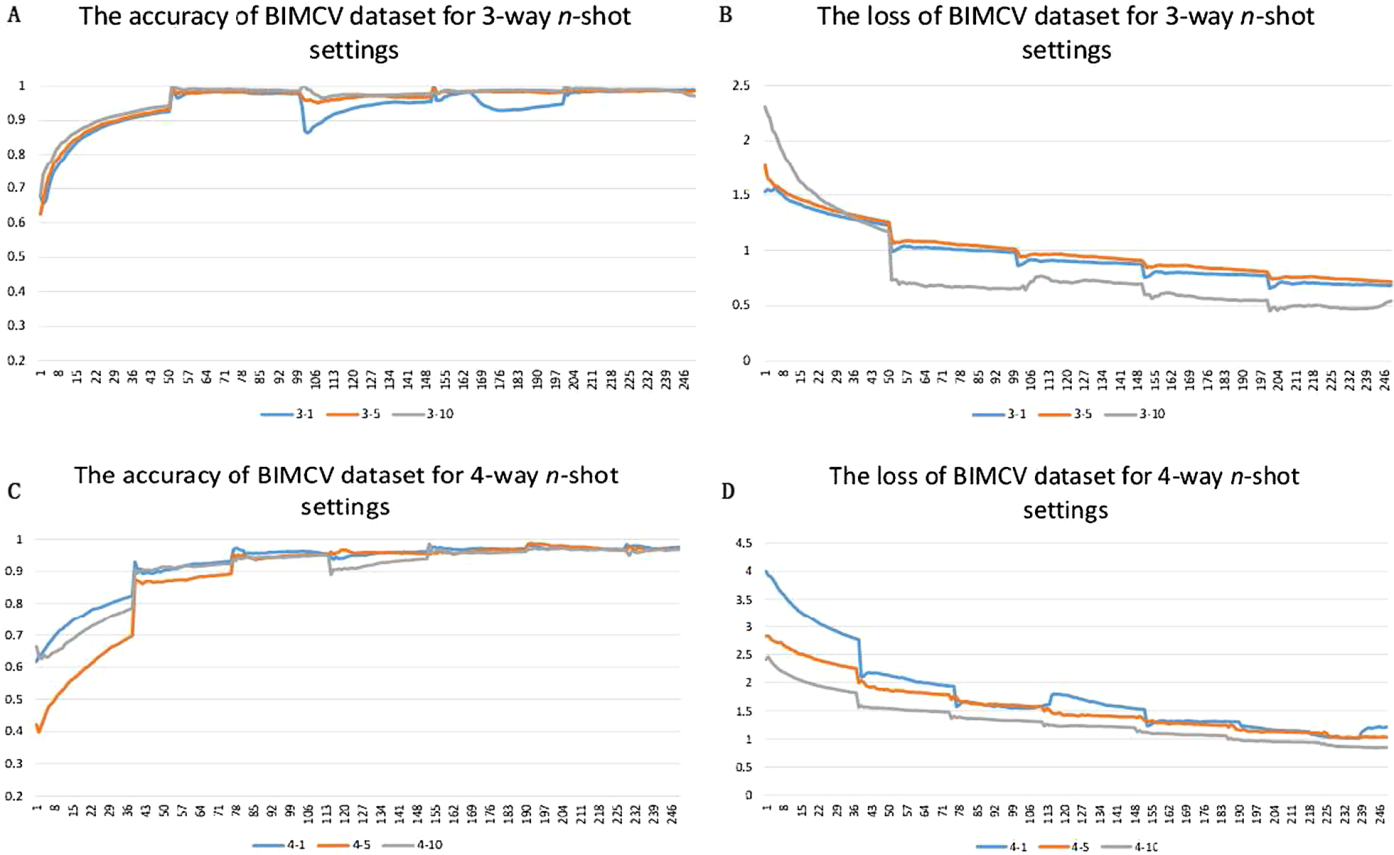
Training accuracy and loss for the 3-way and 4-way DIMCV datasets. The blue line is 1-shot, the orange line is 5-shot and the grey line is 10-shot

**Fig. 6:**
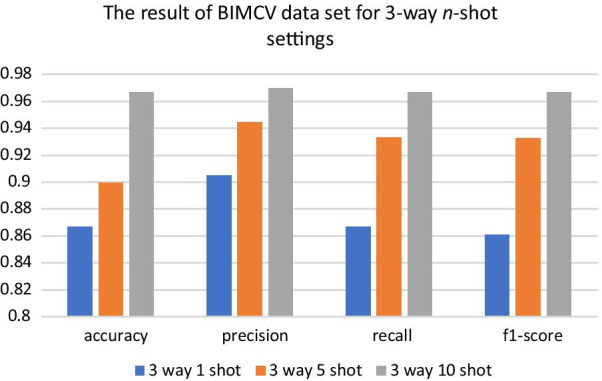
Four indicators of the test dataset results in three-classification tasks

**Fig. 7:**
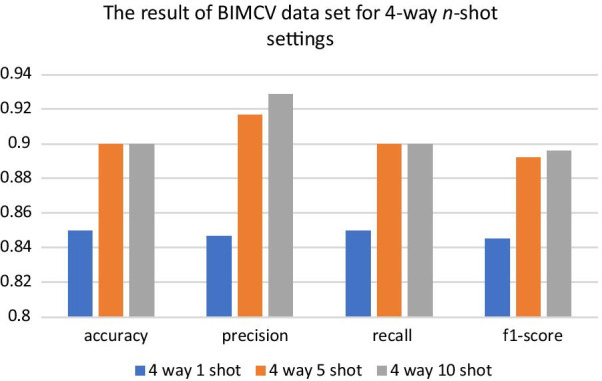
Four indicators of the test dataset results in four-classification tasks

Because UMLF-COVID is a few-shot generalized learning model, it will randomly select k images to train in each epoch. This paper first tested the effect of k-shot, involving 1-shot, 5-shot, and 10-shot methods. Figure 5 shows the value changes of these strategies, and the loss of the model gradually decreases and stabilizes. Figures 6, 7 and Tables 2, 3 show the results of the three and four classifiers of UMLF-COVID. It is worth noting that the accuracy of the model increases with increasing k-shots, while the four classifiers have similar results. With an increased number of shots, the model exploits the benefit of more available pairs of images where it has to distinguish a similar image from different images.

**Table 2:** Details of the results in three-classification tasks with 3-way 10-shot

	Precision	Recall	f1-score
COVID-19	1	1	1
Normal	0.91	1	0.95
Other pneumonia	1	0.9	0.95
Accuracy	0.97	0.97	0.97
Macro avg	0.97	0.97	0.97
Weighted avg	0.97	0.97	0.97

**Table 3:** Details of the results in four-classification tasks with 4-way 10-shot

	Precision	Recall	f1-score
Bacteria	0.83	1	0.91
COVID-19	1	1	1
Normal	0.83	1	0.91
Virus	1	0.6	0.75
Accuracy	0.9	0.9	0.9
Macro avg	0.92	0.9	0.89
Weighted avg	0.92	0.9	0.89

**Fig. 8:**
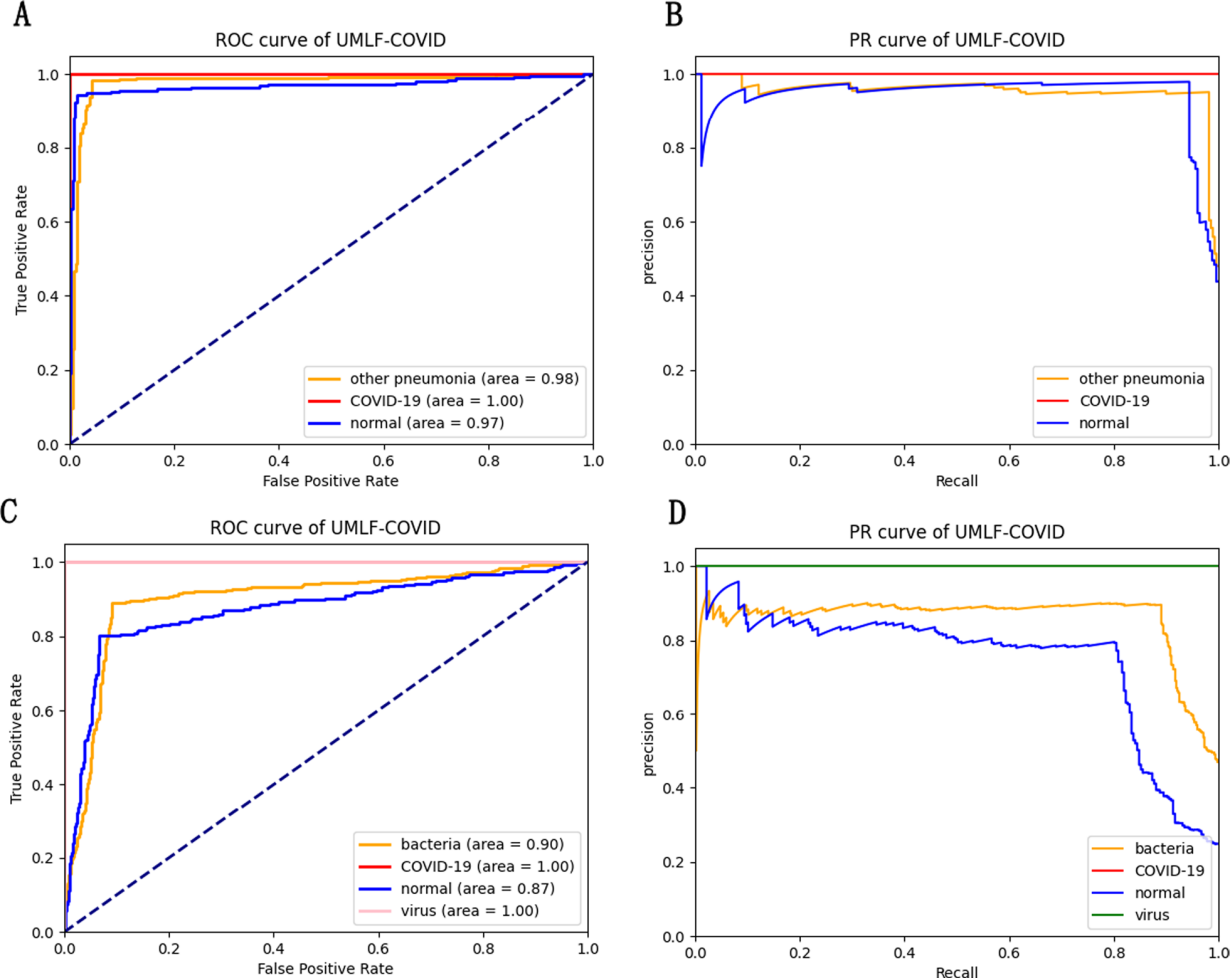
ROC and PR analysis of UMLF-COVID of 10-shots. **a**, **b** are the results of 3-way. **c**, **d** are the results of 4-way analysis, and the lines of COVID-19 and virus overlap

The experiment also compares the n-way 10-shot strategy of UMLF-COVID with other CNN classification models. Because COVID-19 is also a kind of viral pneumonia, COVID-19, normal, and virus classes are retained in the three-classifier. This model also randomly selects three classes from the whole dataset for each epoch. Table 4 shows the classification accuracy results. With the 10-shot strategy, UMLF-COVID obtains the best result at approximately 97%. Compared with the CNN-LSTM model, the best of the compared models, UMLF-COVID shows a 4% accuracy improvement, while the LeNet, Alexnet, VGG, CovXNet CNN-RNN, and EMCNet models only reach 92%, 89%, 83%, 89%, 92%, and 91% accuracy, respectively. Moreover, the precision, recall and F1-score indicators of UMLF-COVID reach nearly 97%. The ROC curve and PR curve are also used to show the performance of models. As shown in Fig. 8 and Additional file 2: Figures S1–S7, the UMLF-COVID model is expected to perform well and is better than the other comparison methods. Especially in COVID-19 patients, the recognition rate can reach 100%.

**Table 4:** The performance of UMLF-COVID and comparison models in three-classification tasks

Model	Precision	Recall	F1-score	Accuracy
UMLF-COVID	**0.97**	**0.97**	**0.97**	**0.97**
LeNet	0.92	0.92	0.92	0.92
Alexnet	0.91	0.89	0.89	0.89
VGG	0.85	0.83	0.83	0.83
CovXNet	0.91	0.89	0.88	0.89
CNN-LSTM	0.93	0.93	0.93	0.93
CNN-RNN	0.92	0.92	0.92	0.92
EMCNet	0.92	0.91	0.92	0.91

The strategy is 3-way 10-shot

Bold values are highlight our model

Finally, this paper retains the COVID-19, normal, virus and bacteria classes to test the models in four-classification tasks. Although four-classification tasks are more difficult than three-classification tasks, the UMLF-COVID model can also obtain 90% accuracy, which is much better than that of other models, and all metrics also present good results (Table 5). The precision, recall and F1-score reach nearly 92%, 90%, and 89%, respectively. However, the accuracy of other compared models only reaches 65–88%, and the precision, recall and F1-score of CNN-LSTM and CovXNet reach 88%. As shown in Fig. 8 and Additional file 2: Figures S1–S7, similar to the three-classification task results, the UMLF-COVID model is superior to the other comparison methods. It can still reach 100% for COVID-19 patients.

**Table 5:** The performance of UMLF-COVID and comparison models in four-classification tasks

Model	Precision	Recall	F1-score	Accuracy
UMLF-COVID	**0.92**	**0.9**	**0.89**	**0.9**
LeNet	0.72	0.71	0.69	0.71
Alexnet	0.71	0.72	0.70	0.72
VGG	0.77	0.78	0.77	0.78
CovXNet	0.88	0.88	0.88	0.88
CNN-LSTM	0.88	0.88	0.87	0.88
CNN-RNN	0.85	0.81	0.79	0.81
EMCNet	0.84	0.84	0.84	0.84

The strategy of is 4-way 10-shot

Bold values are highlight our model

### Results of the XRay_AI dataset

**Fig. 9:**
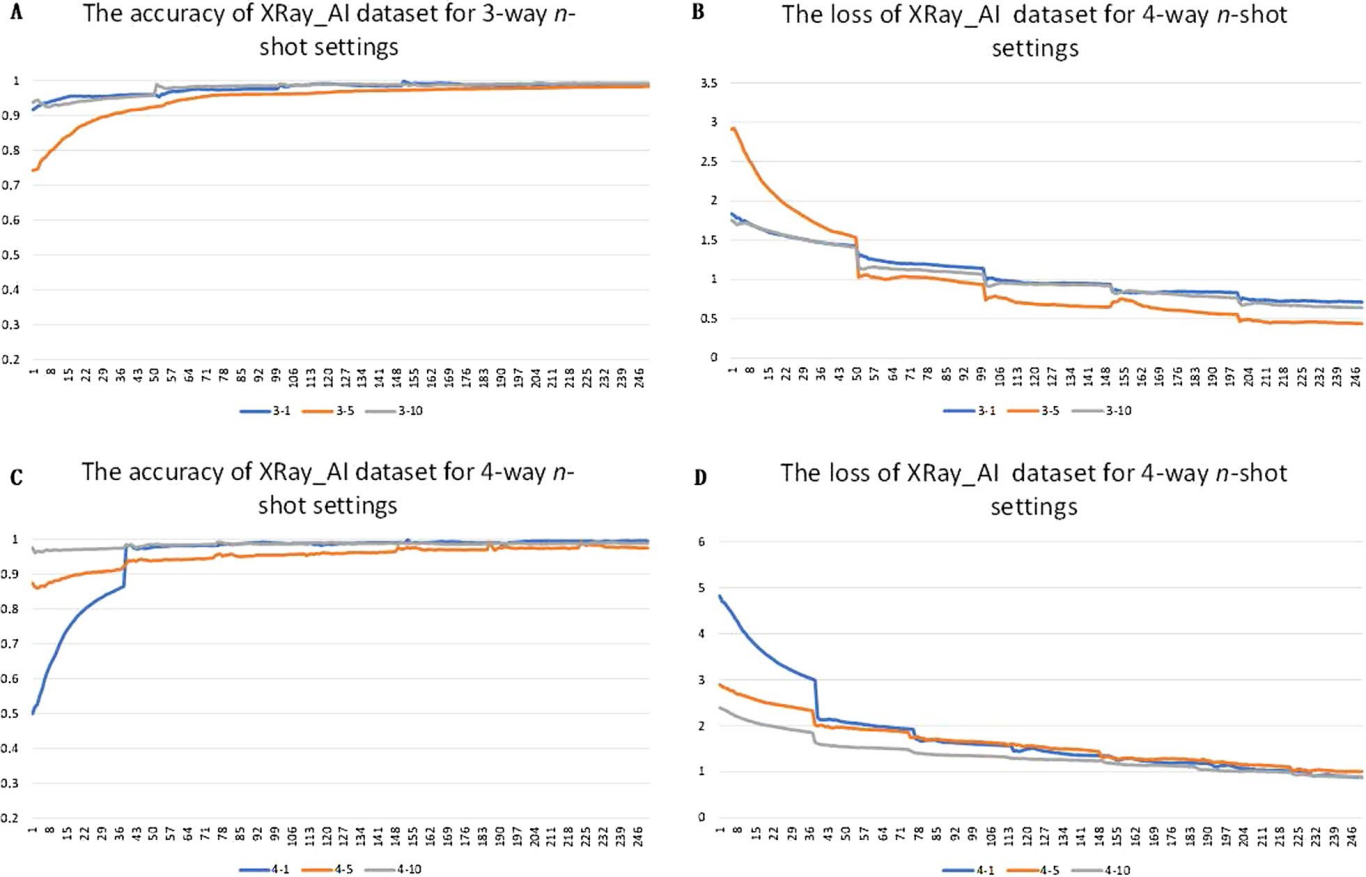
Training accuracy and loss for the 3-way and 4-way XRay_AI datasets. The blue line is 1-shot, the orange line is 5-shot and the grey line is 10-shot

**Fig. 10:**
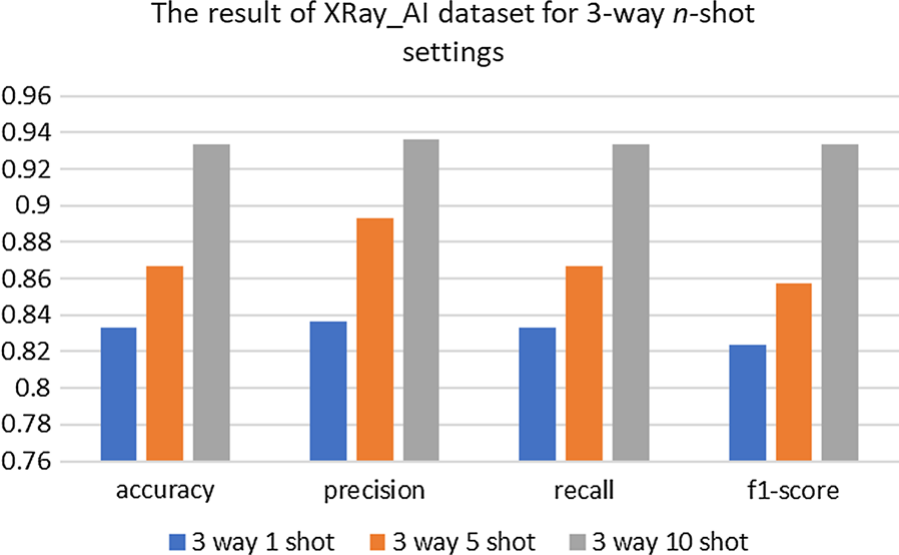
Four indicators of the test dataset results in three-classification tasks

**Fig. 11:**
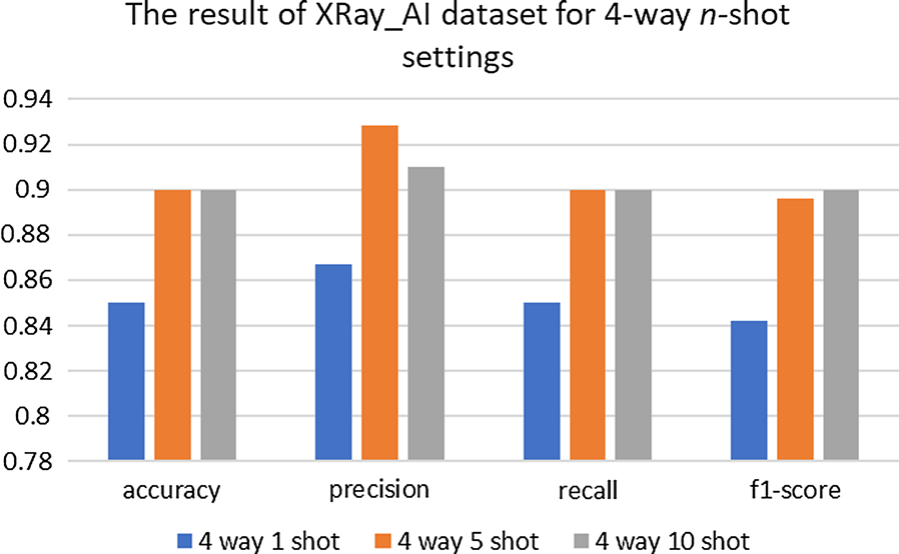
Four indicators of the test dataset results in four-classification tasks

The XRay_AI dataset used different batches of pneumonia diseases: COVID-19, normal, virus, bacteria, and other pneumonia. First, the results of UMLF-COVID with different strategies are showed in Fig. 9. The accuracy of the three-classification tasks is higher than that of the four-classification tasks. With increasing k-shot parameter k, the accuracy is higher; the details of the results are shown in Tables 6, 7 and Figs. 10, 11.

**Table 6:** Details of the results in three-classification tasks with 3-way 10-shot

	Precision	Recall	F1-score
COVID-19	0.9	0.9	0.9
Normal	1	0.9	0.95
Other pneumonia	0.91	1	0.95
Accuracy	0.93	0.93	0.93
Macro avg	0.94	0.93	0.93
Weighted avg	0.94	0.93	0.93

**Table 7:** Details of the results in three-classification tasks with 4-way 10-shot

	Precision	Recall	F1-score
Bacteria	1	1	1
COVID-19	0.83	1	0.91
Normal	1	0.8	0.89
Virus	0.8	0.8	0.8
Accuracy	0.9	0.9	0.9
Macro avg	0.91	0.9	0.9
Weighted avg	0.91	0.9	0.9

**Fig. 12:**
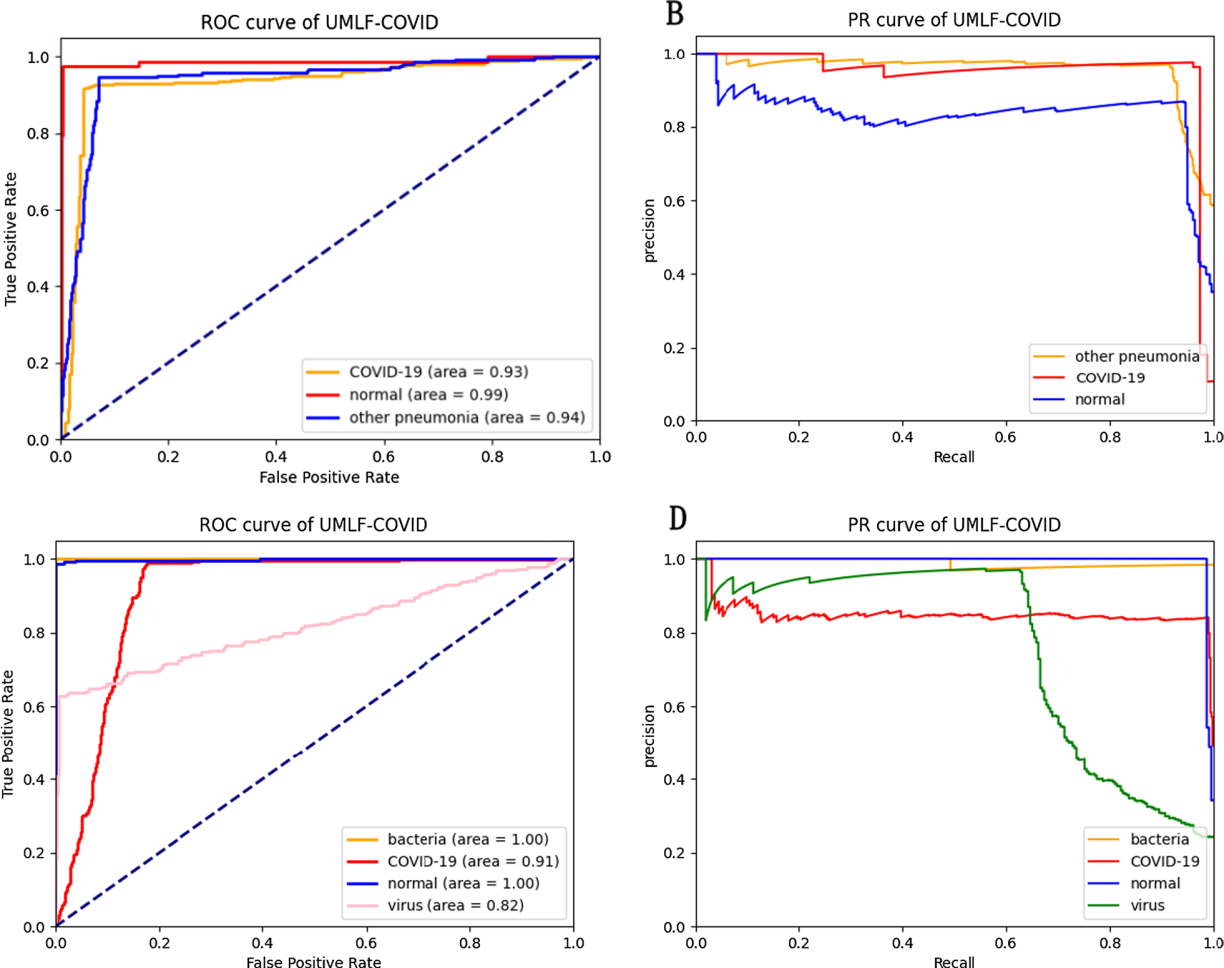
ROC and PR analysis of UMLF-COVID of 10-shots. **a**, **b** are the results of 3-way. **c**, **d** are the results of 4-way, and the lines of bacteria and normal overlap

In the three-classification tasks, the accuracy of UMLF-COVID reaches 93%, and the precision, recall and F1-score are 94%, 93%, and 93%, respectively. The best performing model is CNN-LSTM; its accuracy is 90%, and the precision, recall and F1-score only reach 0.9, 0.9 and 0.9, respectively (Table 8). In addition, according to Fig. 12 and Additional file 2: Figures S8–S14, UMLF-COVID still performs well on the XRay_AI dataset, which is better than the comparison model.

**Table 8:** The performance of UMLF-COVID and comparison models in three-classification tasks

Model	Precision	Recall	F1-score	Accuracy
**UMLF-COVID**	**0.94**	**0.93**	**0.93**	**0.93**
LeNet	0.87	0.81	0.82	0.81
Alexnet	0.9	0.89	0.88	0.89
VGG	0.83	0.72	0.72	0.72
CovXNet	0.83	0.79	0.80	0.79
CNN-LSTM	0.9	0.9	0.9	0.9
CNN-RNN	0.89	0.89	0.89	0.89
EMCNet	0.89	0.89	0.89	0.89

The strategy is 3-way 10-shot

Bold values are highlight our model

The experiment also tests the XRay_AI dataset in four-classification tasks, and the UMLF-COVID model has obvious improvements. The accuracy of UMLF-COVID reaches 90%, and the precision, recall and F1-score are 91%, 90%, and 90%, respectively. CNN-LSTM was the best performing model in the comparison models; its accuracy reached 80%, and the precision, recall and F1-score were 80%, 80%, and 80%, respectively (Table 9). According to Fig. 12 and Additional file 2: Figures S8–S14, the ROC curve and PR curve of UMLF-COVID on the XRay_AI dataset are better than those of the comparison model.

**Table 9:** The performance of UMLF-COVID and comparison models in four-classification tasks

Model	Precision	Recall	F1-score	Accuracy
**UMLF-COVID**	**0.91**	**0.9**	**0.9**	**0.9**
LeNet	0.77	0.77	0.77	0.77
Alexnet	0.78	0.78	0.78	0.78
VGG	0.74	0.73	0.68	0.73
CovXNet	0.74	0.73	0.72	0.73
CNN-LSTM	0.8	0.8	0.8	0.8
CNN-RNN	0.79	0.79	0.79	0.79
EMCNet	0.83	0.79	0.81	0.8

The strategy is 4-way 10-shot

Bold values are highlight our model

In summary, it can be seen from these two datasets that although UMLF-COVID is an unsupervised model, its performance is better than that of existing classification models. Specifically, as the number of task classes increases, the improvement is more pronounced.

## Discussion

X-ray imaging has many advantages in clinical applications, such as low cost and less damage to patients. However, the characteristic of X-ray images is that when encountering a blocked part, the film will not be exposed, and the part will appear white after imaging. This issue creates greater requirements for the accurate judgement of clinicians. Inexperienced clinicians may find it difficult to accurately judge whether a patient has a potential COVID-19 infection based on X-ray images. However, the CT images will pass through the human body in layers, which means that the CT scan can observe the patient's lungs hierarchically, containing more information. This means that it is more difficult to establish a patient identification model for COVID-19 based on X-ray images than for datasets based on CT images. However, considering the convenience of X-ray, it is necessary to establish a patient identification model for COVID-19 based on X-ray images. At present, there are two main limitations to the existing deep learning model, which is based on COVID-19 X-ray images. First, the number of COVID-19 X-ray images is small, which means that the distribution of the training dataset is very unbalanced. Therefore, it is challenging to construct a supervised deep learning model. Second, most deep learning models to process COVID-19 images are based on transfer learning. This method is limited by the reference dataset, and the parameters are difficult to adjust. Therefore, this paper proposes the UMLF-COVID model, which is the first to use an unsupervised meta-learning method to process COVID-19 images. This model does not need pre-training, and it only needs a small number of samples in each cycle, which solves the problem of sample quality and data imbalance in the COVID-19 X-ray dataset. Moreover, we use artificial labels to solve the limitation on the number of categories.

The UMLF-COVID model was discussed with radiologists at the Macau University of Science and Technology Hospital. Radiographers have a positive attitude towards using the UMLF-COVID model to diagnose COVID-19 X-ray data. They believe that the use of big data and intelligent detection (AI) can increase the detection rate of lesions, increase the speed of reporting, and reduce the workload of doctors. However, at the same time, the final detected lesions still require doctors to combine comprehensive clinical analysis to draw accurate conclusions.

## Conclusion

In summary, this paper proposes a COVID-19 X-ray image classification model based on unsupervised meta-learning. The experiment shows that the UMLF-COVID model is better than the existing deep learning models. In two datasets, the performance of three-classification tasks is better than comparison models by 4–14%, and the performance of four-classification tasks is better by at most 17%. The results are satisfactory, but the UMLF-COVID model still has some problems. For example, the UMLF-COVID model can determine whether a patient is a COVID-19 patient but cannot distinguish between mild and severe cases. This idea is very important for clinicians and has higher requirements for the model. In the future, the UMLF-COVID model will be further expanded to improve the multi-class accuracy of the model and enable the model to distinguish between mild and severe COVID-19 patients.

## Supplementary Information

**Additional file 1:** Supplementary Table 1.

**Additional file 2:** Supplementary Materials.

## Data Availability

All datasets used in this article are publicly available online. A Python package of the UMLF-COVID model is available at https://github.com/mr1528126360/UMLF_COVID. The web links of three datasets is available at BIMCV-COVID19 + . https://osf.io/nh7g8/. Chest X-Ray Images. https://www.kaggle.com/paultimothymooney/chest-xray-pneumonia. Chest XRay_AI. https://miracle.grmh-gdl.cn/chest_xray_ai/.

## Abbreviations

CT: Computed Tomography
COVID-19: Coronavirus disease 2019
ARDS: Acute respiratory distress syndrome
RT–PCR: Reverse transcription-polymerase chain reaction
UMLF-COVID: Unsupervised meta-learning recognition model for COVID-19 X image detection
CXR: Chest X-ray images
CNN: Convolutional neural network
LSTM: Long short-term memory
RNN: Recurrent neural network
ROC: Receiver operating characteristic
PR: Precision-recall
