# Supplementary material for: UMLF-COVID: an unsupervised meta-learning model specifically designed to identify X-ray images of COVID-19 patients
Source: BMC Med Imaging. 2021 Nov 22;21:174. doi: 10.1186/s12880-021-00704-2 (PMC8607405; doi:10.1186/s12880-021-00704-2)
Supplement: Supplementary file 2 — Additional file 2: Supplementary Materials. [file 12880_2021_704_MOESM2_ESM.docx]

Supplementary Materials for

**UMLF-COVID: An unsupervised meta learning model specifically designed to identify X-ray images of COVID-19 patients**

${Rui Miao}^{\dagger}$1, ${X\mathrm{in}\mathrm{Dong}}^{\dagger}$1, Sheng-Li Xie2, Yong Liang1,3, Sio-Long Lo*4

1. Institute of Systems Engineering, Macau University of Science and Technology, Avenida Wai Long, Taipa, Macau, China.

2. Guangdong-Hong Kong-Macao Joint Laboratory for Smart Discrete Manufacturing, Guangzhou 510006, China

3. Department of State Key Laboratory of Quality Research in Chinese Medicines, Macau University of Science and Technology, Avenida Wai Long, Taipa, Macau, China

4. Faculty of Information Technology, Macau University of Science and Technology, Avenida Wai Long, Taipa, Macau, China

$\dagger$ These authors have contributed equally to this work

**Supplementary Figure S1**

**Supplementary Figure S2**

**Supplementary Figure S3**

**Supplementary Figure S4**

**Supplementary Figure S5**

**Supplementary Figure S6**

**Supplementary Figure S7**

**Supplementary Figure S8**

**Supplementary Figure S9**

**Supplementary Figure S10**

**Supplementary Figure S11**

**Supplementary Figure S12**

**Supplementary Figure S13**

**Supplementary Figure S14**

Supplementary Figure S1 ROC and PR analysis of Alexnet in BIMCV data set.

Supplementary Figure S2 ROC and PR analysis of CNN-LSTM in BIMCV data set.

Supplementary Figure S3 ROC and PR analysis of CovXNet in BIMCV data set.

Supplementary Figure S4 ROC and PR analysis of LeNet5 in BIMCV data set.

Supplementary Figure S5 ROC and PR analysis of VGG in BIMCV data set.

Supplementary Figure S6 ROC and PR analysis of CNN-RNN in BIMCV data set.

Supplementary Figure S7 ROC and PR analysis of EMCNet in BIMCV data set.

Supplementary Figure S8 ROC and PR analysis of Alexnet in XRay_AI data set.

Supplementary Figure S9 ROC and PR analysis of CNN-LSTM in XRay_AI data set.

Supplementary Figure S10 ROC and PR analysis of CovXNet in XRay_AI data set.

Supplementary Figure S11 ROC and PR analysis of LeNet5 in XRay_AI data set.

Supplementary Figure S12 ROC and PR analysis of VGG in XRay_AI data set.

Supplementary Figure S13 ROC and PR analysis of CNN-RNN in XRay_AI data set.

Supplementary Figure S14 ROC and PR analysis of EMCNet in XRay_AI data set.
